# Malaria and trypanosome transmission: different parasites, same rules?

**DOI:** 10.1016/j.pt.2011.01.004

**Published:** 2011-05

**Authors:** Laura C. Pollitt, Paula MacGregor, Keith Matthews, Sarah E. Reece

**Affiliations:** 1 Institute of Evolutionary Biology, School of Biological Sciences, University of Edinburgh, UK, EH9 3JT; 2 Institute of Immunology and Infection Research, School of Biological Sciences, University of Edinburgh, UK, EH9 3JT; 3 Centre for Immunity, Infection and Evolution, School of Biological Sciences, University of Edinburgh, UK, EH9 3JT

## Abstract

African trypanosomes produce different specialized stages for within-host replication and between-host transmission and therefore face a resource allocation trade-off between maintaining the current infection (survival) and investment into transmission (reproduction). Evolutionary theory predicts the resolution of this trade-off will significantly affect virulence and infectiousness. The application of life history theory to malaria parasites has provided novel insight into their strategies for survival and reproduction; how this framework can now be applied to trypanosomes is discussed. Specifically, predictions for how parasites trade-off investment in survival and transmission in response to variation in the within-host environment are outlined. An evolutionary approach has the power to explain why patterns of investment vary between strains and during infections, giving important insights into parasite biology.

## Protozoan parasites: life history trade-offs

Protozoan parasites, such as African trypanosomes (*Trypanosoma brucei* sp.) and malaria parasites (*Plasmodium* sp.), cause serious mortality and morbidity in humans, livestock and wildlife and have severe economic impacts in the developing world. These parasites undergo asexual replication within a vertebrate host and must produce specialized transmission stages to be transmitted between hosts by insect vectors. Evolutionary theory predicts that this life cycle results in a trade-off between the investment of resources into survival (replication) and reproduction (production of transmission stages; Box 1). Survival versus reproduction trade-offs are a key concept in evolutionary biology and have received a wealth of theoretical and empirical attention [1]. Whereas most of the concepts of life history theory have been developed for multicellular organisms, parasites face similar challenges; species competing for resources within a host and being targeted by the immune response are analogous to prey species competing for food and avoiding predators [2,3]. The predictions of theory are being met with increasing support across a diverse range of taxa [4,5], including single-celled parasites [4,6–8].

In recent malaria research, life history theory has provided insight into how parasites respond to selection pressures, such as co-infection with other genotypes or species, or attack from anti-malarial drugs [4,9–11]. This framework has been successful in explaining the patterns observed in laboratory experiments with model systems [11–14], and there is also some evidence that these findings are relevant to human malaria parasites in natural infections [15] and *in vitro* studies [16]. By contrast, trypanosome research has largely remained focused on molecular and cellular biology (but see [17]). The success of using life history theory to understand the strategies of malaria parasites suggests that this framework can also be applied usefully to trypanosomes to explain variation in parasite strategies, across genotypes and during infections. This article discusses how predictions from life history theory can be applied to understand the investment strategies of trypanosomes. The trade-off between investment in survival (replication) and reproduction (production of transmissible stumpy forms) is focused on for two reasons. First, there are clear and useful parallels with recent findings in malaria parasites (Box 2). Second, the relative investment in within-host replication and between-host transmission is predicted to have significant effects on virulence and infectiousness [18].

## Trypanosomes: survival and reproduction

**Figure 1:**
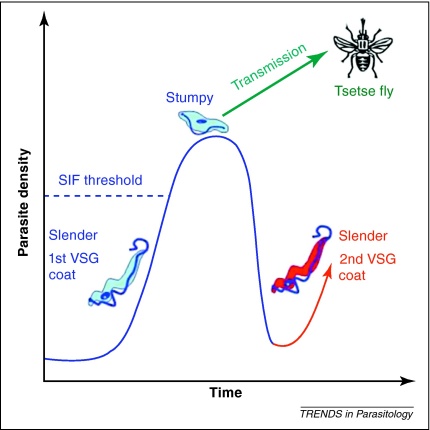
Dynamics of trypanosome infection in the mammalian host. As slender form parasites replicate in the blood, the parasitaemia rises, as does the concentration of a soluble stumpy induction factor (SIF), inducing some parasites to differentiate into non-replicating, but transmissible, stumpy forms. A combination of differentiation into stumpy forms and clearance, as the immune system mounts a response to the first VSG coat, leads to a crash in parasitaemia. However, because some slender forms have switched VSG coats, a second wave of parasites, not yet recognized by the immune system, begins to increase parasitaemia once again.

When an infected tsetse fly bites a mammalian host, metacyclic forms are inoculated into the blood. These develop into slender form parasites that undergo rapid asexual replication, maintaining the infection in the host (survival). As parasite density increases, a parasite-derived factor accumulates (termed stumpy induction factor, SIF) and causes some, but importantly not all, parasites to undergo cell cycle arrest and differentiate into stumpy forms [19]. Stumpy forms have a limited life expectancy in the blood because they no longer replicate or productively switch their VSG coat, but they are infective to tsetse flies and therefore provide the potential for transmission (reproduction) [20]. Trypanosome infections generally involve cyclical peaks in parasitaemia (Figure 1). Statistical modelling indicates that parasite driven differentiation, together with antigenic variation, can generate this pattern, and therefore the distinctive waves of parasitaemia are predominately under parasite control [21]. The role that differentiation plays in generating waves of parasitaemia is supported by observations that laboratory strains that cannot produce stumpy forms continue to replicate, quickly killing the host [22], and that cycles of parasitaemia are still observed in infections of immunocompromised mice [23].

However, in natural infections, parasitaemia will be shaped by a combination of the host immune response and the production of stumpy forms. To evade the host immune response, trypanosomes employ a strategy of changing their variant surface glycoprotein (VSG) surface coat. Each parasite has a repertoire of thousands of VSG genes but expresses only one at a time [24]. Initially in laboratory infections, one or a few VSG variants dominate but the immune system eventually raises antibodies against these coats, leading to wide-scale clearance. Each parasite has a low probability of switching to the expression of a new variant [25]. Therefore, during every replication cycle, a small number of parasites probably have a VSG coat not yet recognized by the immune system, and these parasites will rapidly replicate, resulting in a new wave of parasitaemia (Figure 1) [25]. It is important to note that although variants differ in the VSG gene(s) being expressed over the course of a single infection, they are isogenic to the original infecting parasite clone(s). This is significant because natural selection acts at the level of the parasite genotype within infections, therefore clonally related parasites will be selected as a group to maximize the transmission of their genotype over the course of the infection [7,26].

Each trypanosome faces a trade-off between differentiation into a transmissible stumpy form and continued division as a slender form. From the perspective of a parasite cohort, continued replication of slender forms is necessary to withstand attack from the immune system; for example, maintaining parasite numbers provides the potential to express new VSG coats, whereas stumpy forms provide the potential for between-host transmission. This trade-off has obvious parallels with gametocyte production in malaria parasites (Box 2). Also, similar to malaria parasites, trypanosomes will experience variation in their within-host environment, both during infections and in different hosts, which is predicted to influence the balance between investment in slender and stumpy forms.

## Strategies to maximize survival and reproduction: evolutionary predictions

**Figure 2:**
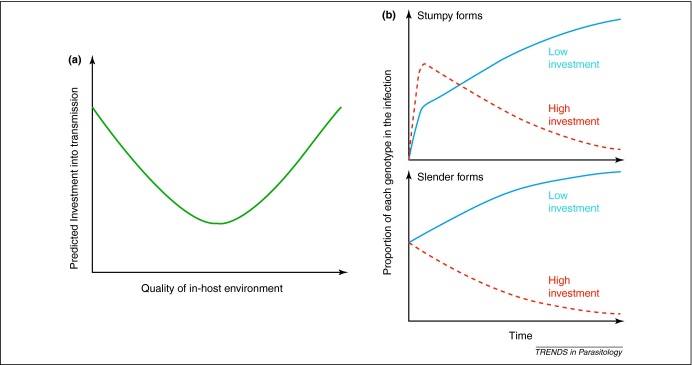
Strategies for the relative investment into transmission stages. **(a)** Theory predicts that organisms will invest heavily in reproduction under either very good or exceptionally poor conditions, and be constrained to investing in survival in intermediate situations [1]. When applied to trypanosomes, parasites are predicted to produce high numbers of transmissible stumpy forms in extremely good or extremely poor within-host environments, but, in most conditions be constrained to producing enough slender form parasites to maintain the current infection. As with malaria parasites, it is probable that there will be genetic variation between strains for the ability to accurately detect and respond to environmental cues, and the level of stress experienced in a given environment [12]. **(b)** When parasites are in mixed infections, differing levels of investment into stumpy forms will influence competitive outcomes. Higher investment in transmission stages (high investment; red dashed line) gives short-term benefits (higher initial rate of transmission) but is detrimental to longer-term success because it is more vulnerable to being cleared. The optimal strategy depends on the duration of infection (chance of being cleared by the immune response or outcompeted and risk of host death) and transmission opportunities for the parasite. For example, in a prolonged mixed genotype infection of trypanosomes, the strain with low investment (blue solid line) has higher fitness because it can transmit for longer.

Trypanosome parasites reach a threshold before some parasites differentiate into transmissible stumpy forms. Evolutionary theory predicts that, in general, the relative level of investment into reproduction will depend on the quality of the environment and that this relation will be U-shaped [1]. For trypanosomes, investment into transmission (differentiation into stumpy forms) should depend on the quality of the within-host environment. Investment in stumpy forms is predicted to be highest under extremely good conditions, when parasites can afford to invest heavily, or extremely poor conditions, where survival is unlikely and parasites employ a strategy of terminal investment. Between these two extremes, parasites will be constrained by investing in within-host survival by adopting reproductive restraint (Figure 2a). The strategies observed at the extremes make intuitive sense, but explaining why reproductive restraint is predicted is more complex. When parasites experience stressful (but not terminal) situations, they must produce enough slender replicating forms to maintain the infection, which lowers the density of transmissible stumpy forms in the short term but maximizes fitness over the course of the infection (Figure 2b). The importance of within-host survival is often overlooked but safe guarding future transmission will be an important determinant of parasite fitness when infections persist over long periods.

## Adding ecology

For trypanosomes, like other parasites, key variables determining the quality of the within-host environment include: exposure to immune responses, availability of host resources, exposure to trypanocidal drugs, and the presence of competitors. Trypanosomes live freely within the circulation and generate energy through glycolysis of blood glucose. Although the occurrence of hypoglycaemia, at least at peak parasitaemia, is indicative of it being a limiting resource, the effect of glucose level on trypanosome development *in vivo* is yet to be quantified. Similarly, if the efficacy of drugs (where applied) or the force of attack by the immune system varies, trypanosomes will be exposed to different levels of stress. Competitive suppression has been demonstrated to occur in trypanosomes [17], and clear parallels can be drawn with the responses of malaria parasites to competitors, which are discussed below. In reality, the overall quality of the within-host environment and the net level of stress parasites experience, is likely to be influenced by interactions between these variables and further complicated by both host and parasite factors. However, as a starting point to develop clear predictions that can be tested with laboratory experiments, it is useful to consider these different stresses in terms of where they will place parasites on the axis of environmental quality (Figure 2a).

## Within-host competition

Like most organisms, parasites (in genetically mixed infections) encounter competitors, and understanding how this affects parasite traits is receiving attention [4,6,10]. Trypanosomes in mixed infections are suppressed, resulting in lower parasite densities [17]. This could be driven by either resource limitation, mixed infections triggering stronger host defenses, or direct interference competition between strains [10,17,27]. Increasing investment in replication could ameliorate competitive suppression by enabling parasites to exploit the greater share of host resources and/or the generation of new VSG variants. Evolutionary theory for malaria parasites predicts that reproductive restraint maximizes competitive ability [28] and a recent laboratory study reveals that they employ this strategy (but see [29]) when in competition [12].

The extent of reproductive restraint parasites should adopt is predicted to depend on the extent of suppression, which is determined by relative competitive ability. For a poor competitor, a mixed infection is likely to be a very bad environment because proliferation is heavily suppressed, and a terminal investment could be the best strategy. By contrast, reproductive restraint might be unnecessary for the best competitors who experience the least suppression. These predictions are consistent with observations that malaria parasites with faster replication rates compete more effectively in experimental mixed infections [30] and can be complicated if competitive ability depends on who the competitors are. Furthermore, postponing transmission in the short term to improve competitive ability could be risky if mixed infections are particularly virulent and will probably kill the host rapidly. However, because natural infections of malaria parasites and trypanosomes are usually chronic and persist over multiple replication cycles and competition suppresses overall parasite density, safeguarding future transmission is likely to be an important component of parasite fitness.

## Complex within-host environments

Importantly, the quality of the within-host environment will be shaped by multiple interacting factors and will vary over the course of infection. For example, parasite interactions between strains are complex, spanning from facilitation to competitive suppression. These interactions will also influence and be influenced by factors including host immunity and resource availability [10,27,31]. Additionally, intrinsic host factors will also be important, for example, the rate of SIF turnover or immune competence might vary between individual hosts, leading to complex feedbacks with parasite strategies. The relative importance of different factors, such as competition, immunity and resource limitation, in shaping the quality of the within-host environment, and thus precisely where they place parasites on Figure 2a, is yet to be determined. A combination of using mathematical models to explain experimental data and developing evolutionary theory specifically for trypanosomes will be extremely useful.

## Responding to environmental change

Parasite investment strategies can be fixed, plastic or a combination of both. Whether parasites evolve fixed or plastic responses to cope with changes in the circumstances experienced during infections depends on: the frequency of encountering situations, the benefits of responding, and the costs and the constraints involved (Box 1) [4,32]. For example, for parasites to plastically alter strategies in mixed infections they must be able to gather information on the genetic diversity of the infection. Bacteria coordinate group behaviours using quorum sensing to transmit and receive information about density and relatedness [33]. Malaria parasites also appear to be capable of responding to density and relatedness, although the mechanism is not yet known [12,14]. Trypanosomes detect and respond to SIF in a density-dependent manner, and there is also evidence for the coordination of group motility behaviours in the tsetse infective (procyclic) form [34].

Given these observations and the extent that the within-host environment varies during infections and between hosts, plastic responses are probable. Trypanosomes could plastically alter investment into stumpy forms by adjusting the amount of SIF produced or their threshold for responding to SIF. Although SIF is yet to be identified [35], experimental work has indicated that it is a small soluble molecule secreted by the replicating slender stages [19]. Adjusting the concentration of circulating SIF could be complicated by variation in rates of host clearance, and whether SIF initiates a response that is strain-specific or pan-infection. Conditioned media produced by one strain was found to be able to induce stumpy form production in two other strains [19], suggesting that SIF could be general across genotypes. Therefore, varying the threshold for responding to SIF might be a better strategy because it could protect parasites from manipulation by co-infecting strains. Laboratory adapted strains become insensitive to the SIF they produce [19]; however, it is not yet known if there is a range of sensitivities or whether it is an ‘all or nothing’ response.

In parasite populations where mixed infections and the resulting competitive suppression are the norm, reduced investment in transmission is likely to become fixed. This could have dramatic effects on virulence to the host: less virulent strains could actually reduce harm by suppressing more virulent strains [17]. But, as demonstrated for malaria parasites, if these virulent parasites are released from competition (for example, by being the only genotype transmitted, or through selective drug treatment), the brakes would be removed from the replication of the virulent strain and hosts would experience more severe disease [11,15,36].

## Where do we go from here?

Life-history theory can provide testable predictions for trypanosome investment strategies. However, to move forward it is necessary to perform controlled and rigorous experiments that examine parasite strategies under manipulated (perturbed) within-host conditions. Because there are clear predictions for how parasites will respond to competition, and mixed infections are a relatively simple experimental manipulation to perform, within-host competition is a good starting point. The integration of mathematical modelling approaches, with experimental data from these experiments, will be crucial to improve our understanding of the complex interactions within infections and their effect on parasite investment strategies. Mathematical models can tease apart the factors and processes underlying biological patterns to form hypotheses that can be tested empirically [37].

## Determining the ecology of mixed infections

There has been little work to quantify the prevalence of mixed infections in trypanosome populations, or their influence on parasite phenotypes. However, field research indicates that there is a range of population structures in African trypanosomes [38,39], as well as genetic variation for traits underlying virulence [40]. The genetic tools available for *Trypanosoma brucei* [41] and large-scale field projects examining the incidence and epidemiology of trypanosome infections could provide a much clearer picture of mixed infections. This requires developing markers to identify, and ideally quantify, different strains. Whereas the ultimate aim will be to understand how the presence of competing genotypes influences trypanosome life history traits and dynamics in natural infections, the first step, as with malaria parasites, will be to perform controlled lab experiments. To do this it will be necessary to increase the number of genetically characterized strains available for experiments. Field strains are available for trypanosomes but are underexploited in experimental settings in favour of laboratory-adapted strains, which although useful for molecular studies, might not provide realistic information on transmission strategies [42].

## Quantifying investment into transmission stages

The development of genotype and stage specific qRT-PCR for malaria parasites has made it possible to track focal genotypes during experimental infections to quantify their investment decisions [43,44]. For trypanosomes, classification of cells as slender or stumpy has traditionally depended on their morphological characteristics, an unreliable process because of the existence of intermediate forms. However, a gene array named PAD (proteins associated with differentiation) involved in transmission has recently been identified [45]. Because PAD marks the transmissible stumpy form, assays to quantify its expression will allow researchers to reliably monitor levels of differentiation over the course of the infection [35]. By comparing patterns of investment in transmission stages of focal parasite genotypes, in single and mixed infections, it will be possible to test for plastic responses to competition. Yet, because transmission investment is predicted to be simultaneously influenced by multiple factors (e.g. competition, resource availability, immune responses) as well as variation in their effects on different parasite genotypes, it is important to measure or control for the effects of potentially confounding variables when examining patterns [4]. To understand dynamics in mixed infections it will also be necessary to examine variation in the response to SIF produced by clone mates and other strains across a range of genotypes from areas where mixed infections are common. Again, controlled experiments will be the first step before analysis of samples from natural infections.

## Conclusions

Evolutionary ecology can explain parasite traits and uncover strategic (adaptive) patterns in what often seems to be noisy data [4,7]. Trypanosomes provide exciting opportunities for integrating evolutionary biology with parasitology. Because much of their molecular biology is well understood, and there are highly tractable tools for reverse genetic analysis, the mechanisms underpinning parasite traits, such as kin discrimination, can be relatively straightforward to identify. In this way, research into trypanosome life-history strategies can feed back into malaria research where these mechanisms are not yet understood. By explaining parasite life-history traits it will be possible to gain insight into how, when, and why traits underlying transmission and virulence vary, which will lead to better informed control strategies [6].

## Figures and Tables

**Table I:** Malaria parasite transmission strategies and the within-host environment

Malaria species	Data source	Environmental change	Predicted level of stress, quality of within-host environment	Effect on relative investment in transmission	Ref.
*P. chabaudi*	Experimental infections in mice	Increased resources	Low stress, high quality within-host environment	All six strains studied increased investment in transmission with higher proportions of young red blood cells (reticulocytes) and five of the six and with total red blood cell density.	[12]
*P. falciparum*	Cultures with drug sensitive strains from natural infections with frequent drug treatment	Exposure to low doses of anti-malarial drugs	Intermediate	Decreased investment in transmission for all three susceptible strains studied.	[16]
*P. chabaudi*	Experimental infections in mice	Presence of conspecific competitor	Intermediate	Decreased investment under competition for all three of the strains studied.	[12]
*P. chabaudi*	Experimental infections in mice	Presence of conspecific competitor	Intermediate	Only significant effect was for decreased investment, but this was only observed in one of two host strains for one of two parasite strains	[29]
*P. chabaudi*	Experimental infections in mice	Exposure to erythropoietin, which signals host anaemia	High stress, low quality within-host environment	Increased investment seen in one strain of *P. chabaudi* but not in one strain of *Plasmodium vinckei.*	[52]
*P. chabaudi*	Experimental infections in mice	Exposure to high doses of anti-malarial drugs	High stress, low quality within-host environment	Increased investment in both of the two strains studied.	[53]
*P. falciparum*	Cultures of laboratory strains	Exposure to high doses of anti-malarial drugs	High stress, low quality within-host environment	Increased investment seen across all four strains studied.	[54]

## Box 1 Key concepts in evolutionary ecology

### Life history trade-offs

Life history traits are phenotypic components influencing fitness [46]. For parasites this includes replication rate and the relative investment into and timing of producing transmission stages [4]. Whereas organisms are selected to maximize fitness, they are also constrained by trade-offs between different life history traits [46]. Trade-offs can take various forms but the most commonly considered result from resource limitation: organisms have limited resources to invest in different life processes and therefore need to balance investment to maximize fitness [46]. In metazoans, single organisms are easily identifiable as the target of natural selection. In single-celled parasites, a genotype within an infection is the comparable target [7,26]. When infections consist of a single genotype, trade-offs will be resolved across all parasites in the host, maximizing overall fitness over the lifetime of the infection [7]. An important trade-off, especially in long lasting infections such as with malaria and trypanosome parasites, is between current investment in between-host transmission and investment in maintaining the infection (within-host survival) for future transmission [4,28].

### Phenotypic plasticity and fixed strategies

Examining and explaining trade-offs is complicated due to organisms evolving under varying environmental conditions. The best solutions to resource allocation trade-offs depend on the opportunities and constraints offered by the within-host environment and how they change throughout infections [4,32].

Environmental conditions can lead to changes in life-history traits by two distinct, but not mutually exclusive, processes [32,47]. First, organisms might be able to produce a range of phenotypic responses according to variation in environmental or internal conditions. This process, known as adaptive phenotypic plasticity, is central to understanding the effects of environmental variation on evolution and can be broadly defined as a change in the phenotype of a given genotype in response to environmental cues [48]. This enables organisms to respond rapidly to predictable environmental changes in ways that maximize fitness [32]. For example, the freshwater crustacean *Daphnia pulex* produces costly morphological defences, including neck spines, when exposed to predator cues [49]. Second, with longer-term environmental changes, spanning multiple generations, microevolution can occur where population gene frequencies change because of individuals best adapted to the new conditions disproportionably contributing to future generations [32]. For example, when a new high coverage drug treatment is introduced, genes for resistance mechanisms spread in the population of parasites targeted [50].

Plasticity and microevolutionary processes can work together to shape the genotypes and the phenotypes organisms display [32,48]. Organisms that are likely to encounter a range of environmental conditions use plasticity to match their phenotype to changes in their circumstances. But maintaining mechanisms to detect, process, and respond to environmental cues is costly and organisms also risk inaccurate cues leading to the wrong phenotype [4,51]. For these reasons different degrees of plasticity will occur in natural infections, and if environmental changes stabilize, phenotypic plasticity may be replaced by fixed strategies [4].

## Box 2 Malaria parasite investment strategies in response to stress

Malaria parasites replicate asexually within the red blood cells of their vertebrate host but also produce specialized transmission stages (gametocytes). When male and female gametocytes are taken up in a mosquito blood meal they produce gametes and undergo a round of sexual reproduction to infect the vector. The trade-off faced by malaria parasites between asexual replication and production of gametocytes is analogous to the growth versus reproduction trade-off faced by all sexually reproducing organisms [4]. Getting the right balance is essential; too few transmission stages results in an evolutionary dead end within the host, but too many can lead to the infection being cleared before a transmission opportunity arises.

Mounting evidence from experimental *Plasmodium chabaudi* infections in mice and *in vitro* cultures of *Plasmodium falciparum* show that malaria parasites vary their investment in gametocytes during infections depending on aspects of their environment (Table I). These patterns initially seem contradictory and confusing but can be explained in a life history framework in which parasites are expected to respond to changes in the constraints and opportunities experienced during infections [4]. Malaria parasites increase investment in gametocytes when experiencing either very good or very poor conditions. When conditions are good (e.g. high density of preferred red blood cells), parasites have plenty of resources and can afford to invest in gametocytes [12,52]. When conditions are very poor (e.g. high drug doses or severe resource limitation), then continued survival in the host is unlikely, and parasites make a terminal investment in gametocytes to maximize short-term transmission [53,54]. More commonly, parasites experience intermediate stress levels (e.g. competition with other strains, low levels of drugs or host immune factors), and are constrained to ensuring within-host survival by reducing investment in gametocytes (reproductive restraint) [12,16,28]. It is important to note, however, these patterns will be made more complex by the details of interactions between host and parasite genotypes [29].

A life history framework not only explains variation observed in malaria transmission strategies [12,14,16], but these studies have also provided novel insights into other aspects of their biology. The ability to respond to various aspects of their within-host environment reveals parasite mechanisms to detect information about their surroundings. Most strikingly, malaria parasites respond to both the genetic diversity of their infection and the densities of their own genotype and co-infecting con-specifics [12,14]. This demonstrates an ability to discriminate between kin and non-kin previously thought limited to complex multicellular organisms.
